# Unraveling Jawbone Susceptibility: Distinctive Features Underlying Medication-Related Osteonecrosis

**DOI:** 10.3390/dj14010018

**Published:** 2026-01-01

**Authors:** Balázs Paczona, József Piffkó, Ágnes Janovszky

**Affiliations:** Department of Oral and Maxillofacial Surgery, University of Szeged, Kalvaria 57, H-6725 Szeged, Hungary; paczona.balazs.patrik.02@szte.hu (B.P.); piffko.jozsef@med.u-szeged.hu (J.P.)

**Keywords:** jaw, long bones, bone regeneration, bone metabolism, medication-related osteonecrosis of the jaw, macrophages

## Abstract

Medication-related osteonecrosis of the jaw (MRONJ) is a devastating complication arising primarily after invasive dentoalveolar procedures in patients treated with antiresorptive, antiangiogenic, or targeted therapies. Although recognized risk factors are established, the distinctive vulnerability of jawbones compared to long bones is not fully understood. This review comprehensively synthesizes recent advances regarding the embryological, anatomical, and physiological disparities that contribute to region-specific susceptibility to MRONJ. Recent evidence suggests that jawbones diverge significantly from long bones in embryonic origin, ossification pathways, vascular architecture, innervation patterns, and regenerative capacities. These differences affect bone metabolism, healing dynamics, response to pharmacologic agents, and local cellular activities, such as enhanced bisphosphonate uptake and specialized microcirculation. Experimental and clinical evidence reveals that mandibular periosteal cells exhibit superior osteogenic and angiogenic potentials, and the jaws respond differently to metabolic challenges, trauma, and medication-induced insults. Furthermore, site-specific pharmacologic and inflammatory interactions, including altered periosteal microcirculation and leukocyte–endothelial interactions, may explain the development of MRONJ, although rare cases of medication-related osteonecrosis have also been reported in long bones. Emerging research demonstrates that immune dysregulation, particularly M1 macrophage polarization with overexpression of matrix metalloproteinase-13 (MMP-13), plays a crucial role in early MRONJ development. Understanding these mechanisms highlights the critical need for region-specific preventive measures and therapeutic strategies targeting the unique biology of jawbones. This comparative perspective offers new translational insights for designing targeted interventions, developing tissue engineering solutions, and improving patient outcomes. Future research should focus on gene expression profiling and cellular responses across skeletal regions to further delineate MRONJ pathogenesis and advance personalized therapies for affected patients.

## 1. Introduction

Marx RE published the first report on the potential adverse effects of third-generation bisphosphonates (BISs) in 2003 [1]. However, further clinical observation and studies confirmed the role of antiresorptive therapy alone or in combination with immune modulators or antiangiogenic medications in the pathomechanism of this severe disorder, resulting in the change in nomenclature from bisphosphonate- to medication-related osteonecrosis of the jaw (MRONJ) [2]. Recent studies have expanded the understanding of MRONJ pathogenesis beyond traditional antiresorptive agents, with new evidence linking sclerostin inhibitors like romosozumab to osteonecrosis development [2]. Despite the known drug- or patient-related risk factors potentiating the development of MRONJ (e.g., invasive dentoalveolar procedure, indication and duration of the therapy, or administration route), the exact pathomechanism has not yet been clarified [2]. In general, experimental and clinical research investigated the potential role of local inflammatory or infectious processes, and the cytotoxic or impaired regenerative effects of these drugs [3,4,5,6,7]. Contemporary research has identified immune dysregulation as a central mechanism, with specific focus on altered macrophage polarization and compromised host defense mechanisms in the jawbone environment [8]. Interestingly, special features or skeletal differences explaining the vulnerability of the jawbones, which may also contribute to the development of MRONJ, have not been summarized. This narrative review synthesizes comparative evidence on jawbones and long bones in the context of medication-related osteonecrosis of the jaw (Table 1). The literature search was performed primarily in PubMed/MEDLINE, complemented by manual screening of reference lists. The main search terms included combinations of “jaws”, “mandible”, “maxilla”, “long bones”, “bone regeneration”, “bone metabolism”, “bisphosphonate” (or specific medication), “denosumab”, “antiangiogenic therapy”, “medication-related osteonecrosis of the jaw”, “immune dysregulation”, “macrophage polarization”, “periosteum”, and “periosteal microcirculation”, mainly focusing on experimental and clinical studies that directly compared jaws with long bones or provided insight into MRONJ pathogenesis, with a particular emphasis on embryological, anatomical, vascular, neural, cellular, and immunological differences, while case reports and small case series were generally excluded.

## 2. Embryological, Anatomical, and Physiological Features of the Jawbones

### 2.1. Embryological Differences

A crucial difference can be observed between flat and long bones, namely in the process of bone formation. Flat bones, such as craniofacial bones, are characterized by *intramembranous ossification*. This type of bone formation is typical in bones developing from the neural crest. During intramembranous ossification, the embryonic mesenchyme forms a collagen membrane containing osteochondral progenitor cells. These cells differentiate into osteoblasts, a cell type responsible for bone formation [9]. Osteoblasts form ossification centers, secreting an intercellular matrix creating a scaffold for later occurring mineralization. Calcium is then bound by the matrix, entrapping osteoblasts and leading to their transformation into osteocytes. Osteocytes play an important role in bone remodeling and in mineralization processes locally and systemically [9,10,11]. Hypoxia-induced neovascularization in the central parts contributes to the formation of spongious bone, while externally mesenchymal cells differentiate to periosteal cells [12]. Adjacent cells to this layer are newly forming osteoblasts, which contribute to the development of cortical bone [13]. Recent evidence suggests that intramembranous ossification involves more complex mechanisms than previously understood, with osteochondrogenic progenitors co-expressing Sox9 and Runx2 transcripts within developing intramembranous bones, creating a “chondroid” bone phenotype that combines rapid proliferation with mineralization capacity [14].

Instead, long bones undergo *endochondral ossification*, a process where embryonic mesenchymal stem cells differentiate into prechondrogenic mesenchymal cells. Through intercellular signaling, cell–cell adhesion is increased, resulting in the formation of condensation. Subsequently, supplying blood vessels regress, creating a hypoxic environment within the central chondrogenic condensate [9]. The hypoxia-induced signaling pathway activates mesenchymal neovascularization [15]. Following the condensation phase, chondrocyte differentiation occurs. Initially, the condensate is surrounded by the perichondrium, which restricts perpendicular growth, allowing only elongation. Later, chondrocytes transition into a hypertrophic state, preparing the bone for the ossification phase [14]. Terminally hypertrophic chondrocytes release signaling molecules, including VEGF, which activates chondroblasts, resulting in channel-like resorptions in the cartilage. Vascularization of these channels occurs, leading to osteoblast activation and the onset of ossification. The perichondrium transforms into periosteum, as osteochondral progenitor cells within the periosteum differentiate into osteoblasts. Blood vessels and osteoblasts of the newly formed periosteum invade the calcified cartilage template [9,16]. Internally, osteoblasts form spongy bone at primary ossification center, while externally, periosteal osteoblasts form compact bone. In summary, load-bearing cartilage is replaced and rebuilt into the bone [17].

It is worth noting that the embryonic origin of the craniofacial skeleton differs from that of the bones of the extremities. While the former originates from the neural crest, the latter are of mesodermal origin. This distinction plays a crucial role in the osteogenic capabilities of the aforementioned bones [14]. Recent lineage tracing studies have revealed that neural crest-derived cells maintain aspects of their pluripotency program through transient reactivation of Oct4 and Nanog, which may contribute to their enhanced regenerative capacity [18]. Osteoblasts derived from the neural crest exhibit higher activation from FGF proliferative signaling compared to those of mesodermal origin [19]. These differences are evident during embryonic stages, resulting in higher osteogenic marker expression, more intense bone mineralization, and larger bone nodule formation in frontal bone-derived osteoblasts. Additionally, these cells may influence nearby cells in a paracrine fashion, promoting cell growth and osteoblast differentiation [20].

### 2.2. Particular Anatomical Features of the Bones

Bones of the human body are complex, constantly changing structures. The general composition of bones can be divided into an inner collagenous spongious bone and an outer dense, cortical bone, which is covered by the periosteum [21]. The periosteum consists of two different layers, with distinct roles: an outer fibrous layer and an inner more vascular and cellular layer, known as the cambium layer. The outer part of the fibrous layer is a significant contributor to the blood supply of bone and skeletal muscle and contains a rich neural network, while the deeper part of the fibrous layer (fibroelastic layer) is cell-poor, barely vascularized, and responsible for periosteal tendon attachments [13,22]. The inner or cambium layer plays a crucial role in the metabolism of the skeletal structures and new bone formation or growth. This is owing to the high cellularity, which includes mesenchymal progenitor cells, differentiated osteogenic progenitor cells, osteoblasts, and fibroblasts [21,22]. Furthermore, rich peripheral vascular and neural sympathetic networks are presented in the cambium layer. As age advances, the cambium undergoes a progressive decrease in thickness, vessel density, and regenerative potential, eventually becoming indistinguishable from the outer fibrous layer [22,23]. The continuous blood supply of the bones is essential to ensure physiological bone remodeling, metabolism, and regeneration; however, this is one of the most important differences between jawbones and long bones. While the long bones receive their vascular supply from the nutritive arteries, the circulation of the jaws is provided by the mucoperiosteal tissue [24,25,26,27,28]. This fundamental difference in vascular architecture has significant implications for MRONJ pathogenesis, as the jaw’s dependence on mucoperiosteal circulation makes it more vulnerable to the antiangiogenic effects of medications [29]. Moreover, not only the blood supply but also the periosteal innervation and its patterns show certain differences between jawbones and long bones. The mandibular nerve provides the innervation of the lower third of the maxillofacial region, but in a unique way, it contains both afferent and efferent nerve fibers [30]. While neural networks traverse across the surface of the mandible, the tibial periosteum exhibits a longitudinal orientation. Vasoactive intestinal polypeptide-positive nerve fibers form small networks with individual fine varicose fibers in the mandibular periosteum, whereas larger networks are to be seen at the tibia. These fine fibers are associated with both vascular and nonvascular elements, suggesting specific functions in the mandibular periosteum [31]. During the fetal development, the appearance and density of calcitonin gene-related peptide (CGRP)-positive nerves (primarily localized to C and Aδ sensory fibers), which play a role in the neurogenic inflammatory processes, also show differences in the mandible compared to the tibia [32]. Moreover, the density of CGRP-positive nerves in the mandible increases toward the mandibular canal, and interestingly, toward the periodontal ligament from periosteum [33]. A comparative in vitro study investigating mandibular and tibial periosteal cells revealed site-specific differences favoring the mandible. Mandibular periosteal cells exhibited superior osteogenic, angiogenic, and endogenous potential, as well as enhanced activation of FGF signaling, compared to tibial periosteal cells, while the calvarial periosteum has lower osteogenic potential than the tibial periosteum [20,34,35,36]. Recent studies have confirmed these findings and demonstrated that jawbone periosteum-derived cells (jb-PDCs) maintain high osteogenic and chondrogenic potential, with enhanced expression of bone regeneration markers compared to long-bone periosteal cells [37].

Cadaver studies also uncovered structural disparities among bony tissues. Specifically, the trabecular structure of the mandible exhibited plate-like formations, whereas a combination of plate- and rod-like structures was evident in the tibia, and only rod-like trabecular structures were presented in the ilium. In addition, significant differences in bone mineral density and bone volume/total volume were observed in the lower jaw compared to the tibia or ilium [38]. These parameters also showed variations among anatomical regions of the mandible or jawbones and are influenced by tooth loss (dentulous or edentulous jaw). In the edentulous mandible, the trabecular structure transitioned from plate-like to rod-like patterns [38,39,40,41,42]. Furthermore, it has been found that the microcirculation of the jaw features a higher number of anastomoses and a greater impact of the centromedullary circulation as opposed to the long bones [43]. This difference in vascular structure may influence how the jaw responds to injuries and treatments, contributing to its unique healing and regenerative properties.

In addition to structural differences, bones exhibit distinct signaling properties and cellular activities depending on their origin [44]. Studies have shown that bone marrow stem cells or osteoblasts of the mandible possess a remarkable capacity to induce bone formation both in vitro and in vivo, as well as a higher angiogenic potential compared to long bones, although the expression of the VEGF gene may alter over time [45,46,47,48]. These characteristics may contribute to a higher degree of healing capacity of the mandible after fracture compared to other anatomical locations [47,49,50,51]. Moreover, cartilage-specific proteins or genes responsible for angiogenesis and ossification process showed site specificity, where angiogenic potential was higher in the mandibular condylar cartilage [46,52].

### 2.3. Bone Modeling and Remodeling: Cellular and Intercellular Characteristics

Modeling occurs continuously during skeletal development, greatly reducing and ceasing entirely after skeletal maturity, while remodeling takes place throughout life. Modeling leads to changes in bone shape and size, whereas remodeling generally maintains them. These processes involve two major types of bone cells: the osteoclasts and the osteoblasts [53,54]. Their very special communication, known as osteoclast–osteoblast coupling, is responsible for maintaining bone homeostasis, the balance between bone resorption and formation [54,55]. This complex intercellular communication is regulated by various factors, including cytokines, growth factors, hormones, or cell surface receptors [56]. One of the key signaling pathways involved in osteoclast–osteoblast coupling is the receptor activator of nuclear factor kappa-B ligand (RANKL)-RANK pathway. RANKL, produced by osteoblasts and other bone marrow stromal cells, binds to its receptor RANK on osteoclast precursor cells, leading to their differentiation into mature osteoclasts and their activation for bone resorption [57]. Conversely, osteoblasts secrete osteoprotegerin (OPG), a decoy receptor for RANKL, which competitively inhibits the binding of RANKL to RANK, thereby suppressing osteoclastogenesis and bone resorption. This delicate balance between RANKL and OPG regulates the formation and activity of osteoclasts in response to various physiological and pathological stimuli [58]. Additionally, a mechanism in the opposite direction can be observed, where osteoclast-mediated processes (e.g., TGFβ1 and SMAD3 signaling, or sphingosine-1-phosphate) promote osteoblastogenesis [59]. This cross-talk between osteoclasts and osteoblasts is a fundamental process in bone biology that regulates bone remodeling and maintains skeletal integrity. These fine mechanisms show distinct differences between the jaws and long bones, as the maxillofacial region has unique characteristics such as alveolar bone, tooth eruption, and orthodontic tooth movement. Comprehensive studies investigating the bone marrow of the mandible and long bones, both in vitro and in vivo, have demonstrated a diverse osteoclastogenic and osteogenic capacity in the mandible compared to the long bones [45,60,61]. For instance, certain osteoclast genes (e.g., Nfatc1, Dc-stamp, Ctsk, or Rank) are upregulated in mandibular-derived osteoclast precursors [62]. Additionally, proliferation, alkaline phosphatase activity, and the expression of genes related to bone regeneration, bone growth, extracellular matrix mineralization, and bone remodeling (e.g., osteopontin, Msx) show differences and higher activity in the mandible compared to the long bones [47]. These mechanisms may contribute to shorter bone healing times after fracture or bone augmentation in the mandible compared to the long bones of the extremities [47,63,64].

The role of innervation in bone metabolism is a relatively novel aspect of research. Experimental and clinical studies have revealed the contribution of sensory and sympathetic neuronal systems in bone development, growth, and remodeling [65,66,67]. These nerve fibers, which are primary afferent sensory and sympathetic fibers frequently associated with blood vessels, are present mostly in the periosteum, and less so in bone marrow and mineralized bone [66]. Several neuromediators, such as vasoactive intestinal polypeptide, calcitonin gene-related peptide, pituitary adenylate cyclase activating peptides, neuropeptide Y, substance P, noradrenaline, serotonin, and glutamate, are involved in the regulation of bone cell activity, bone development, and regeneration [66,68,69,70]. Generally, CGRP downregulates osteoclastogenesis and osteoclastic activity by blocking the RANK/RANKL pathway, while substance P has the opposite effect. Both proteins, however, upregulate osteoblast activity and new bone formation, thereby accelerating fracture healing [71]. Interestingly, experimental sympathectomy and sensory denervation do not appear to alter normal bone growth but are involved in local remodeling. Sympathectomy significantly increased the number of osteoclasts on the mandibular bone surface, while sensory denervation resulted in the opposite effect [31,72,73,74].

## 3. Variances in the Skeletal Manifestation of Medical Conditions

Several studies have shown that various diseases, such as osteoporosis and fracture healing, can affect bones differently, with a more rapid onset of osteoradionecrosis often observed in the mandible (Table 1) [75,76,77,78,79]. Factors, such as continuous mechanical loading of the mandible during mastication, unique anatomical and morphological characteristics, and the distinct embryological origin of the jaws and long bones, may contribute to this phenomenon [76,80,81,82]. Dysregulation of osteoclast–osteoblast coupling is implicated in various bone disorders, including osteoporosis, rheumatoid arthritis, and Paget’s disease. Imbalances in bone resorption and formation disrupt skeletal homeostasis, leading to bone loss, fractures, and compromised bone healing.

### 3.1. Effects of Nutrition

Malnutrition affects the function and recovery of every organ system, including bones [83]. In animal models, mandibular alveolar bone was found to be less sensitive to protein undernutrition [76]. Regarding low calcium intake, studies are controversial, showing either no effects or inhibiting mandibular growth [84,85,86,87]. Related to calcium homeostasis, the anabolic role of 1,25(OH)2D did not differ in the investigated anatomical regions (alveolar bone of the mandible and long bones). However, parathyroid hormone exerts an anabolic effect predominantly in long bones, contributing to site-specific differences in PTH receptor and IGF1 expression [88]. An experimental study found that diabetes significantly affects bone structure, with the tibia experiencing the most severe bone loss. In contrast, the femur, mandible, and spine showed less immediate bone loss, with significant decreases observed later. By the third month, the femur, mandible, and spine experienced significant reductions in bone volume/trabecular volume [89].

### 3.2. Osteoporosis

Osteoporosis is characterized by decreased bone mineral density and bone mass, or altered structure and strength of bone, leading to an increased risk of skeletal related events, namely bone fractures. Several risk factors may contribute to the development of osteoporosis or increase its likelihood (e.g., sex, age, body size, race, family history, altered hormonal state, diet, co-morbidities, medications, or life-style) [90,91].

Most experimental studies have been conducted using animal models of osteoporosis induced by steroid treatment or estrogen deficiency. The results in this area are somewhat contradictory: some studies have found significant effects of osteoporosis on the mandibular bone features, while others have reported no or negligible differences between the jaws and long bones in certain parameters investigated [76,77,92,93,94,95,96,97,98]. Oral functions, such as mastication, may provide a protective effect against osteoporosis-related skeletal changes in the jaws [99,100]. Although patients with osteoporosis showed radiologically detectable mandibular changes, this process did not correlate with an increased tendency for tooth loss [101,102,103].

The deteriorative effects of osteoporosis on bone healing are well-known, but the literature also contains contradictory results [104]. Some animal models have shown that intramembranous ossification is more sensitive to osteoporosis, while other studies have not demonstrated a significant effect on the osseointegration of titanium implants in the mandible compared to the long bone [105,106,107]. Human studies have not proven a correlation between osteoporosis and mandibular healing or the loss of dental implants [108,109].

### 3.3. Fracture and Bone Healing

Endochondral and intramembranous fracture healing processes are regulated by a complex interplay of signaling molecules, such as bone morphogenetic proteins (BMPs), transforming growth factor-beta (TGF-β), and vascular endothelial growth factor (VEGF) [110]. These factors ensure the proper recruitment, differentiation, and function of osteoblasts and osteoclasts, which are essential for bone formation and remodeling [111]. The role of periosteal integrity in bone physiology is well-established, extending beyond the maintenance of vascular supply to include the active regulation of bone metabolism and regeneration [112]. Successful fracture healing requires the regeneration of both periosteal and endosteal circulations [113]. Periosteal damage can lead to disrupted bone healing, resulting in delayed union or pseudoarthrosis formation [114,115,116]. Clinical and experimental observations indicate that certain long-term medical treatment or medical conditions can impair angiogenesis in the periosteal tissues, leading to further complications [117,118,119].

Endochondral and intramembranous fracture healing are the two primary pathways through which bone repairs itself after an injury. Both processes are crucial for restoring bone integrity, but they differ in their mechanisms and the types of fractures they primarily address [111]. *Endochondral fracture healing*, typically seen in long bones, involves the formation of a cartilage callus as an intermediary step. When a fracture occurs, an (1) initial inflammatory phase sets in, characterized by the formation of a hematoma at the fracture site. Inflammatory cells release cytokines and growth factors, recruiting mesenchymal stem cells (MSCs) to the injury site. This is followed by (2) cartilage formation with early endochondral ossification and a periosteal response, during which MSCs differentiate into chondrocytes, forming a soft callus made of cartilage. This soft callus stabilizes the fracture and provides a scaffold for new bone formation. As healing progresses, (3) cartilage resorption and primary bone formation begin, and the cartilage undergoes endochondral ossification, where it is gradually replaced by woven bone. Blood vessels invade the area, bringing osteoprogenitor cells that further aid bone deposition. Finally, (4) secondary bone formation and remodeling occur, where the woven bone is remodeled into lamellar bone, restoring the original structure and strength of the bone [111,120,121].

In contrast, *intramembranous fracture healing* occurs primarily in flat bones and does not involve a cartilage intermediate [111]. Instead, it begins directly with the formation of bone tissue from mesenchymal cells. After a fracture, the inflammatory response similarly recruits MSCs to the fracture site. However, these cells differentiate directly into osteoblasts. Periosteum-derived stem cells, which are crucial for bone regeneration, show the highest osteogenic potential in the mandible. In contrast, tibial periosteum and bone marrow stem cells are more effective in chondrogenesis [49,51,122]. Osteoblasts begin secreting bone matrix, which mineralizes to form woven bone. Throughout this process, a rich blood supply is maintained, providing the necessary nutrients and cells for bone formation. The woven bone is then remodeled into lamellar bone, ensuring the restored bone is strong and well-structured [111]. Recent evidence demonstrates that jawbone defects can be repaired through endochondral ossification when appropriate conditions are created, with periosteal-derived cell spheroids maintaining chondrogenic potential and contributing to cartilaginous callus formation [123]. The maxillofacial region is unique in that wound and bone healing occur in a somewhat contaminated environment. Despite this, perioperative antibiotic therapy is generally not recommended following tooth extraction, yet gingival and alveolar bone healing still proceed effectively.

### 3.4. Medication-Related Osteonecrosis of the Jaws: Characteristics of the Bone and Medication Interaction

**Figure 1:**
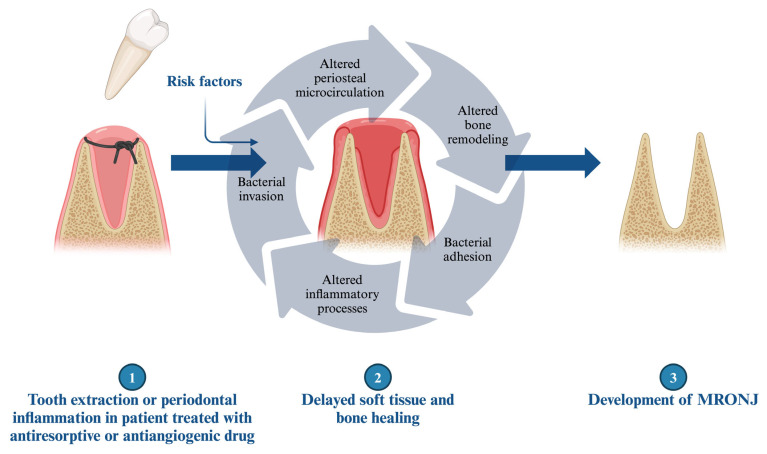
The underlying mechanism in the development of MRONJ.

Medication-related osteonecrosis of the jaw (MRONJ) is a severe complication associated with antiresorptive (such as bisphosphonate and denosumab) or antiangiogenic treatment, with an unclear pathomechanism (Figure 1) [1,2]. These reactions do not typically occur in the bones of the appendicular skeleton [124,125]. Various factors may contribute to or increase the risk of the development of MRONJ, including the administration route, the duration and indication of the therapy, co-morbidities, concomitant drug use, and genetic factors [2,126,127,128,129,130]. However, the primary trigger factor for the development of MRONJ, aside from the aforementioned treatment, is the injury to the alveolar bone, particularly during dentoalveolar procedures [131]. MRONJ predominantly occurs in the molar and premolar regions and is more common in the mandible [132]. This severe condition can develop several years after treatment, which may be explained by the long half-lives of bisphosphonates (BISs) [130]. Recent clinical studies have distinguished between osteoporotic and oncologic MRONJ, revealing that oncologic patients exhibit rapid disease onset, fewer purulent signs, larger sequestra, and lower cure rates compared to osteoporotic patients, suggesting distinct pathophysiological mechanisms between these populations [133].

As previously described, the effects of BIS treatment vary depending on anatomical localization (Table 1). An experimental study investigated the effects of chronic BIS treatment on morphometric indices related to bone quantity and structure. The analysis revealed that quantity-related indices (bone volume/trabecular volume and trabecular thickness) were more impacted in the mandible, while structure-related indices (trabecular pattern factor and trabecular number) were more significant for the femoral epiphysis and metaphysis [134,135]. Another animal study showed that BIS treatment resulted in significant structural changes in the cortical bone channel network of the tibia, with no differences observed in the mandible [136]. Additionally, BIS treatment caused over-mineralization, deterioration in bone mineral quality, decreased proteoglycan content, and deterioration in collagen structural integrity in newly formed bone in the mandible. Despite these effects, mandibular growth was not affected. These adverse effects were not observed in the long bones [137,138]. Additionally, Vieira et al. demonstrated that alendronate may increase intramembranous ossification in the maxillary bone [139].

The regional uptake of BIS is higher in the mandible compared to other skeletal regions, potentially impairing regenerative processes and contributing to the pathophysiology of MRONJ [140]. This preferential accumulation in jawbones is attributed to the higher bone turnover rates in alveolar regions, where bisphosphonates bind to hydroxyapatite crystals and remain sequestered for extended periods [141]. From a functional perspective, bone regeneration relies not only on the activity of the osteoblasts and osteoclasts, but also on the blood supply and angiogenesis. BISs influence all of these processes by primarily inhibiting osteoclast recruitment to the bone surface and shortening their lifespan, either directly or indirectly through the receptor activator of nuclear factor κB (RANK)/receptor activator of nuclear factor κB ligand (RANKL)/osteoprotegerin pathway [142,143]. BIS decreases RANKL levels in the mandible while having the opposite effect in the tibia [6]. Consequently, it can delay bone healing after maxillofacial fracture and decrease bone formation and vascularity in extraction sockets [144,145,146,147]. Numerous studies have demonstrated the antiangiogenic effects of BISs both in vitro and in vivo [144,148,149]. Zoledronic acid, a potent third-generation bisphosphonate, has been shown to inhibit endothelial cell proliferation with IC50 values of 4.1–6.9 μM for various growth factors and to reduce vessel sprouting in multiple angiogenesis assays [148]. Moreover, zoledronate-treated rats exhibited thicker and less connected blood vessels in the alveolar bone of the mandible after tooth extraction [150]. BISs bound to the bone surface can inhibit the growth and proliferation of stem/osteoprogenitor cells in the periosteum. They also exert toxic effects on various cell types, including fibroblasts, osteoblasts, endothelial cells, and epithelial cells [23,151,152,153,154,155,156,157,158,159,160]. As mentioned above, osteoblasts exhibit different functional activities at various locations under physiological conditions, which are critically influenced by BIS treatment [161]. These negative effects can be further exacerbated by the critically high concentration of BIS in the mandible [140,155,162]. These findings may explain their contribution to the lower bone turnover in the mandible and to the development of MRONJ [163,164,165]. Contrarily, in a recently published study, the viability of mesenchymal stem cells from the mandibular or tibial periosteum and bone marrow was not influenced by BIS treatment [166]. Sensory denervation, which plays a distinct role in bone formation as mentioned above, via inferior alveolar nerve transection also facilitated the occurrence of MRONJ in a rat model [71]. Recent mechanistic insights have revealed that MRONJ pathogenesis involves complex immune dysregulation. M1 macrophage polarization with overexpression of MMP-13 plays a crucial role in early MRONJ development, leading to collagen network disruption around affected bone areas. This inflammatory cascade is triggered by decreased defense capacities of the jawbone due to antiresorptive-drug-induced immune suppression and osteoclast inhibition [167].

Special BIS-associated inflammatory changes can be observed in the bone and periosteal microcirculation of the head neck region, and were not seen in long bones [3,7,146,168]. Enhanced leukocyte–endothelial interactions require increased expression of adhesion molecules on the cell surface [169]. However, BISs do not appear to influence the expression of the neutrophil-derived adhesion molecule CD11b, which is responsible for leukocyte adherence. This suggests that endothelial changes may be responsible for the enhanced leukocyte–endothelial interaction localized to the mandibular periosteum [7].

Furthermore, BIS treatment has site-specific impacts during the early healing stages of fractures, delaying callus formation, cartilage development, and bone remodeling specifically in the mandible in a dose-dependent manner [75,147]. The functional activity of osteocytes also differs between the mandible and tibia, with the adverse effects of BISs on bone healing being confined to the jaw, although more bone formation was observed in BIS-treated ovariectomized rats [6,170,171]. Even a single systemic dose of BIS leads to site-specific differences in gene regulation related to tissue healing and bone regeneration. In the tibia, BIS treatment increased proinflammatory cytokines, as well as osteogenic and angiogenic gene activity, whereas in the mandible, the expression of genes related to osteogenesis, inflammation, angiogenesis, bone remodeling, and apoptosis was reduced [172]. BIS pretreatment inhibited the osseointegration of allografts, affecting osteogenesis and resulting in a gap between the allograft and bone surface [173]. Atypical femoral fractures related to chronic BIS treatment have also been documented. However, local use of BIS increased callus volume in femoral fracture healing [174,175,176]. Similar beneficial effects were demonstrated in animal models focusing on osseointegration. Regardless of the local or systemic use of BIS, a single-dose injection promoted the osseointegration of titanium implants in long bones in osteoporotic rats [172,177]. Contemporary understanding suggests that MRONJ development follows an “inside–outside” hypothesis, where persistent bone microdamage from chewing combined with suppressed bone remodeling leads to bone death, and an “outside–inside” hypothesis, where medication-induced immune suppression compromises the oral mucosa’s ability to fight pathogens that eventually spread to underlying bone. The jawbones’ thin mucoperiosteal covering provides minimal protection compared to the thick skin and muscle layers protecting other bones [178].

In summary, multiple theories exist regarding the pathomechanism of MRONJ, yet they all converge on a common factor: altered regenerative processes. One of the most plausible approaches involves changes in the periosteal microvasculature. Medications and related inflammatory reactions can alter the periosteal microcirculation, making the jaws more vulnerable and reducing their regenerative potential. BISs enhance bacterial adhesion (e.g., Pseudomonas, Staphylococci) and biofilm formation on bone hydroxyapatite, exacerbating the risk of infection [179]. In this compromised environment, tooth extraction, impaired regeneration, and delayed wound healing promote further bacterial contamination of the extraction socket or alveolar bone through gingival injuries [180,181]. Recent studies emphasized the prominent role of local infection, and a correlation was found between bacterial colonization (e.g., Porphyromonas, Lactobacillus, Tannerella, Prevotella, Actinomyces, Treponema, Streptococcus or Fusobacterium) and the development of MRONJ [182,183]. Emerging therapeutic approaches focus on modulating the immune response, with studies showing that interventions promoting M2 macrophage polarization (such as rosiglitazone treatment) or inhibiting M1 macrophage activation and pyroptosis by blocking the NF-κB/NLRP3/IL-1β axis can reduce MRONJ burden by reversing the pathological M1/M2 polarization ratio and decreasing both osteonecrosis percentage and bone exposure [184,185]. Furthermore, M1-M2 macrophage polarization status may correlate with the clinical stage of MRONJ [186]. Comprehensively delineating the mechanisms and extent of macrophage involvement in MRONJ pathogenesis will advance our understanding of disease biology and may uncover intrinsic targets for therapeutic intervention.

Regarding skeletal related events, antiresorptive or antiangiogenic treatments have improved the survival rates and the quality of life of the patients. However, despite its low prevalence, MRONJ and aforementioned medications significantly impair the quality of life of the patients by complicating complex dental rehabilitation, including preprosthetic surgeries and dental implantation.

### 3.5. Future Directions and Conclusions

Comparative studies on gene expression profiles, cellular behaviors, and responses to different stimuli across skeletal regions will further elucidate and enhance our understanding of the underlying mechanisms. Recent advances in single-cell sequencing and lineage tracing techniques promise deeper insights into the molecular characteristics of neural crest-derived versus mesoderm-derived bone cells and their differential responses to medications [187].

However, several critical gaps remain. The functional integrity of jaw periosteal microcirculation and neurovascular coupling is another underexplored area. Advanced imaging modalities (e.g., perfusion MRi) could clarify how antiangiogenic and antiresorptive agents compromise periosteal microcirculation and neural regulation, which is key to bone turnover and repair. Similarly, detailed characterization of the immune microenvironment in the jaw during MRONJ onset is still needed. Moreover, the interaction between oral bacteria and drug-altered bone tissue awaits multi-omics characterization. Integrated microbiome, transcriptomics, and metabolomics studies in MRONJ lesions would reveal novel insights into microbial influences driving persistent inflammation and impaired healing.

This accumulated knowledge will support advances in tissue engineering, including biomimetic scaffolds and controlled growth factor delivery systems designed to recapitulate jaw-specific molecular environments. Emerging bio-integrated scaffolds that generate localized hypoxic microenvironments show promise for promoting endochondral ossification and enhanced healing of large bone defects [188]. Understanding the distinct molecular and cellular mechanisms in different skeletal regions can enable personalized medical approaches, optimizing therapeutic strategies based on mandibular or long-bone requirements. Future therapeutic strategies should consider the unique embryonic origins of jawbones by preferentially using neural crest-derived progenitors for mandibular repair over mesoderm-derived cells [189].

Additionally, these insights may contribute to the development of effective preventive and therapeutic approaches for patients with MRONJ. Promising avenues include immune modulation therapies targeting macrophage polarization, exosome-based delivery of bioactive molecules, and combination approaches addressing both the vascular and immune components of MRONJ pathogenesis [190].

Addressing these specific research gaps through focused mechanistic and translational studies will enable region-specific interventions, ultimately improving the clinical management and quality of life of patients at risk of or affected by MRONJ.

## Figures and Tables

**Table 1:** Distinctive differences between jawbones and long bones and their relevance to MRONJ susceptibility.

Aspects/Aspect	Jawbones (Maxilla or Mandible)	Long Bones (e.g., Tibia, Femur)	Suggested Relevance or Implication for MRONJ
**Embryonic origin and ossification**	Predominantly neural crest–derived, mainly intramembranous ossification.	Mesoderm-derived, predominantly endochondral ossification.	Neural crest lineage and intramembranous ossification are associated with distinct progenitor profiles and signaling, possibly altering drug responses and regenerative capacity under antiresorptive/antiangiogenic therapy.
**Periosteal structure and microcirculation**	Thin mucoperiosteum with highly vascular cambium layer; rich anastomoses; blood supply largely from mucoperiosteal vessels.	Thicker soft-tissue envelope; nutrient artery system dominates; periosteum more fibrous.	Medication-induced antiangiogenic and microcirculatory changes may have a greater impact in jaws, where periosteal blood flow is critical and coverage is thin, predisposing to osteonecrosis after dentoalveolar surgery.
**Trabecular architecture and turnover**	Higher turnover, plate-like trabeculae in dentate regions; marked site-specific variability with tooth loss; high remodeling around alveolar bone.	More rod-like or mixed plate–rod patterns; turnover generally lower and less directly exposed to functional loading-induced microtrauma.	Higher baseline turnover and microdamage from mastication, combined with potent remodeling suppression, may lead to accumulation of microcracks and impaired repair in jaws.
**Periosteal and marrow progenitor phenotype**	Mandibular periosteal cells show superior osteogenic and angiogenic potential, distinct gene expression, and enhanced FGF signaling; jawbone-derived cells maintain high osteogenic/chondrogenic capacity.	Tibial periosteum and marrow cells relatively more chondrogenic; periosteum of the calvaria often less osteogenic than jaw periosteum.	Under high local drug concentrations, jaw-specific high-activity progenitor niches may become disproportionately vulnerable, with impaired osteogenesis and angiogenesis worsening healing after extractions or implant surgery.
**Innervation and neuropeptides**	Dense sensory and autonomic innervation; mandibular periosteum with rich networks of CGRP- and VIP-positive fibers; close association with periosteal vessels and periodontal ligament.	Periosteal innervation is more longitudinal and less dense; different distribution of CGRP-positive fibers.	Neurovascular–immune interactions in the jaw periosteum may modulate inflammation and bone turnover in a site-specific manner, amplifying drug-induced immune dysregulation and delayed healing.
**Systemic disease impact (osteoporosis, diabetes, irradiation)**	Often shows earlier or more pronounced changes in some conditions (e.g., osteoradionecrosis, region-specific responses to osteoporosis or diabetes), but with partial protection from masticatory loading in others.	Osteoporosis and metabolic disease classically quantified in long bones; some models show greater structural deterioration in tibia compared with mandible.	Disease–medication interactions may be regionally different; jawbones may reach a “threshold” of compromised vascularity and remodeling under combined systemic and local insults more quickly than long bones.
**Drug distribution and pharmacodynamics**	Higher regional bisphosphonate uptake in mandible; site-specific changes in RANKL/OPG expression and gene regulation; pronounced antiangiogenic and cytotoxic effects in mandibular periosteum and extraction sockets.	Lower local bisphosphonate load: in some models, bisphosphonates increase callus volume and support implant osseointegration in long bones.	Preferential jaw accumulation and distinct gene responses contribute to stronger suppression of remodeling, angiogenesis, and soft-tissue repair in jaws, creating a microenvironment prone to necrosis after dentoalveolar procedures.
**Microbiological/microbiome environment**	Constant exposure to oral microbiota; thin mucosa; frequent microtrauma; MRONJ sequestrate often harbors dense Actinomyces and complex biofilms.	Deeply covered by skin and muscle; sterile environment in health; medication-related osteonecrosis outside jaws remains rare and typically not exposed to oral biofilms.	Drug-compromised bone and mucosa in jaws are directly exposed to oral biofilms; enhanced bacterial adhesion to bisphosphonate-coated bone and chronic infection may maintain inflammation and drive MRONJ progression.
**Immune response and macrophage polarization**	Evidence of early, pronounced M1 macrophage polarization, MMP-13 overexpression, collagen breakdown, and impaired resolution of inflammation in MRONJ lesions.	Comparable mechanisms less frequently lead to clinically exposed osteonecrosis in long bones under similar therapies.	Jaw-specific immune and vascular context may favor chronic M1-dominant inflammation around exposed bone, perpetuating necrosis and inhibiting repair; M2 modulation reduces MRONJ in experimental models.
**Trabecular and cortical microarchitecture under chronic bisphosphonates**	Quantity-related indices (bone volume, trabecular thickness) more affected in mandible; over-mineralization, deterioration in collagen and proteoglycan content, and reduced bone quality observed without clear loss of mandibular growth.	Structure-related parameters (trabecular pattern factor, trabecular number) more affected in femoral regions; cortical channel network of tibia can show significant remodeling under bisphosphonates while mandible remains structurally less altered.	Jawbone microarchitecture becomes denser but more brittle, with impaired capacity to repair microdamage, favoring necrosis when overloaded or surgically traumatized.
**Gene expression response to single or chronic bisphosphonate exposure**	In mandible, reduced expression of osteogenic, angiogenic, remodeling, inflammatory, and apoptosis-related genes after treatment; decreased RANKL levels reported.	In tibia, bisphosphonate may increase proinflammatory, osteogenic, and angiogenic gene activity; RANKL levels can increase rather than decrease.	Opposite gene regulation patterns suggest that the same systemic therapy promotes bone formation and callus in long bones but suppresses healing programs in jaws.
**Periosteal microcirculation and leukocyte–endothelial interactions**	Bisphosphonate-associated inflammatory changes and enhanced leukocyte–endothelial interactions documented in mandibular periosteum; endothelial alterations suspected as drivers of localized microcirculatory dysfunction.	Similar periosteal microcirculatory changes have not been consistently observed in long bones under comparable dosing.	Selective periosteal microvascular injury in jaws may critically impair post-extraction and post-implant healing, contributing to localized osteonecrosis.
**Effects on extraction socket and fracture healing**	Delayed socket healing, reduced bone formation and vascularity in mandibular extraction sites; early fracture healing in mandible is dose-dependently delayed (callus formation, cartilage development, remodeling).	Local or short-term systemic bisphosphonate can increase callus volume and enhance femoral fracture healing; in osteoporotic rat models, a single dose can improve implant osseointegration in long bones.	Procedures that are beneficial or neutral in long bones can be detrimental in jaws, explaining why routine dentoalveolar surgery may trigger MRONJ while long-bone fracture care often benefits from antiresorptive treatment.
**Effects on periosteal and stem/progenitor cells**	Bisphosphonates show cytotoxicity toward mandibular periosteal stem/progenitor cells, osteoblasts, endothelial cells, fibroblasts, and oral epithelial cells; some studies report no effect on viability but altered function.	In long bones, progenitor cell viability and osteogenic support for implants and fractures are often preserved or even improved with carefully dosed bisphosphonates.	Functional impairment of periosteal and mucosal progenitors in jaws hampers soft-tissue closure and bone regeneration at exposed sites, perpetuating necrosis.
**Antiangiogenic therapies and non-jaw osteonecrosis**	MRONJ is the predominant clinical manifestation in the maxillofacial region under antiangiogenic/antiresorptive therapy.	Rare cases of osteonecrosis of femoral head and other long bones reported with anti-VEGF or other antiangiogenic agents, but with much lower incidence compared with MRONJ.	Confirms that systemic agents can induce osteonecrosis at multiple sites, but local anatomy, microcirculation, loading, and microbiome exposure make the jaws the most vulnerable target.
**Implants and osseointegration under antiresorptive treatment**	In mandible/maxilla, chronic high-dose regimens are associated with increased MRONJ risk around implants; impaired osseointegration and healing in extraction/augmentation areas reported in susceptible patients.	In long bones, single- or low-dose bisphosphonate regimens may enhance titanium implant osseointegration and improve fixation in osteoporotic bone.	Demonstrates a region-dependent “therapeutic window”: doses and durations that are beneficial for long-bone implants may simultaneously increase MRONJ risk around dental implants.

## Data Availability

No new data were created or analyzed in this study.
